# Effects of Taekwondo Training on Body Composition: A Systematic Review and Meta-Analysis

**DOI:** 10.3390/ijerph182111550

**Published:** 2021-11-03

**Authors:** Seunghui Baek, Jong-Beom Park, Sang-Hwan Choi, Jae-Don Lee, Sang-Seok Nam

**Affiliations:** 1 Department of Health Exercise Management, Sungshin Women’s University, Seoul 02844, Korea; sh100@sungshin.ac.kr; 2 Taekwondo Research Institute of Kukkiwon, 32 Teheran 7-gil, Seoul 06130, Korea; jb1907@hanmail.net (J.-B.P.); cshtkd@hanmail.net (S.-H.C.); drlee@kukkiwon.or.kr (J.-D.L.)

**Keywords:** Taekwondo, meta-analysis, body composition, obesity

## Abstract

Background: The purpose of this study is to investigate the effect of Taekwondo training on body composition and to evaluate the magnitude of the effect. Methods: Databases were used to select studies related to the effectiveness of Taekwondo training, and the inclusion criteria were as follows. Results: Thirty-seven studies were selected. We found statistically significant differences from the control group in weight, body mass index (BMI), waist circumference (WC), waist–hip ratio (WHR), body fat mass, body fat percentage, lean mass, and muscle mass. Also, the age group was statistically significant in control variables on weight, BMI, and body fat percentage. Conclusions: Taekwondo training had a positive effect on body composition, and these results suggest that Taekwondo training is an effective exercise method to lower obesity.

## 1. Introduction

Insufficient physical activity was reported as the fourth most important risk factor among the causes of death worldwide [1]. For this reason, Korea has set physical activity goals for all age groups through the 3rd National Health Promotion Plan (HP2020) and is creating various policies and environments to promote physical activity [2]. According to the Korea Centers for Disease Control and Prevention report in 2019, the rate of aerobic exercise among adolescents increased by 3.0% *p* from 10.9% in 2009 to 13.9% in 2018. On the other hand, the rate of aerobic exercise among adults over the age of 19 and older decreased by 9.8% from 58.3% in 2014 to 48.5% in 2017, and the rate of walking among adults over the age of 19 and older decreased by 6.7% *p* from 45.7% in 2007 to 39.0% in 2017 [3]. However, according to the study of Guthold et al. [4], a 2016 survey of 298 schools in 146 countries found that 81.0% of 11–17 year olds were not getting enough physical activity. On the other hand, in the case of Korea, 94.2% of adolescents showed insufficient amount of physical activity, which made Korea recognized as the country with the most insufficient amount of physical activity for adolescents in the world. Since such a decrease in physical activity can cause a decrease in physical strength and muscle mass [5] and an increase in body fat mass, thereby causing metabolic diseases [6], efforts to increase physical activity are required.

Taekwondo is a traditional martial art from Korea and is recognized as a global martial arts and sport with more than 100 million practitioners in 210 countries worldwide [1]. Taekwondo as an exercise has positive effects on the psychological and physiological areas for the growth and development of children and adolescents. In addition, Taekwondo training prevents or positively improves obesity, dyslipidemia, diabetes, hypertension, cerebrovascular, and cardiovascular diseases in adults and the elderly [7,8,9,10]. In addition, it is expected to improve various physical strengths, including aerobic capacity, muscle strength, muscular endurance, flexibility, speed, and agility through the physiological effects of Taekwondo practice [11,12,13,14]. For this reason, Taekwondo is considered suitable as an essential exercise for improving the physical activity of Koreans and preventing and improving various diseases. However, although multiple studies have been conducted to verify the effectiveness of Taekwondo training, the results are not consistent, and depend on the study subject, method and duration of interventions, making it difficult to generalize the results. Therefore, it is necessary to present the best evidence to prove the effect of physical activity through Taekwondo among Koreans [15]. Thus, this study shows the effects of Taekwondo training on changes in body composition comprehensively and quantitatively to serve as evidence for the development of Taekwondo training as a war to improve obesity caused by lack of exercise in modern people including not only Koreans but people worldwide.

## 2. Materials and Methods

This study was conducted with reference to the Preferred Reporting for Systematic Reviews and Meta-Analysis (PRISMA) guidelines [16].

### 2.1. Search Strategy

In this study, we searched the literature in electronic databases, including the Cochrane Library, EMBASE, and PubMed, and used RISS (Research Information Sharing Service), NDSL (National Science and Technology Information Center) in Korea. The keyword used in the literature search was “Taekwondo”. In addition, we included gray literature such as dissertations to reduce publication bias. The language of the papers was limited to Korean and English. The publication year was not limited to the default setting. The reason we used only ‘taekwondo’ as a keyword is to prevent the omission of literature related to this topic as much as possible.

### 2.2. Selection of Studies

We selected literature according to the following PICO (participants, interventions, comparisons, outcomes) criteria. We included studies where the study subjects were the general public and excluded studies where the study subjects were not the general public (athletes, disabled people, hypertensive patients, diabetic patients, etc.). In addition, we selected studies where the intervention method was Taekwondo training and excluded studies that added interventions other than Taekwondo. Also, we included only studies with a control group who did not practice Taekwondo. We selected studies that presented actual measurements of body composition and studies with an RCT (randomized controlled trial) study design.

### 2.3. Moderator

The effects of Taekwondo intervention were analyzed by looking at how the results were influenced by the differences in the moderators, including gender, age, training period, research method, types of literature, and quality evaluation. Age was divided into three groups: students who attend elementary, middle, and high school, adults, and the elderly. The training periods were divided into 12 weeks or less and more than 12 weeks. Publication types were identified by dividing them into degree thesis and journal publications.

### 2.4. Quality Assessment

For the qualitative evaluation of the selected literature, the Cochrane Collaboration’s RoB (Risk of Bias) tool [16] was used. The evaluation consists of 7 items, including: (1) generating order of random assignment; (2) hiding assignment order; (3) blinding study participants and researchers; (4) blinding outcome evaluation; (5) insufficient result data; (6) selective reporting; and (7) other biases. The evaluation was rated as low risk, high risk, and uncertain.

### 2.5. Statistical Analysis

The effect size of the Taekwondo intervention and the heterogeneity were analyzed using the R program (version 3.6.2, R Foundation for Statistical Computing, Vienna, Austria) [17]. The outcome variables were all continuous variables and included Height, weight, BMI, WC, WHR, fat mass, lean mass, body fat percentage, and muscle mass. The overall effect size was analyzed when there were three or more outcome variables. The measurement unit was the same, expressed as mean difference (MD) and 95% confidence intervals (Cls). The random effects model was used under the assumption that the outcome variables of each study were heterogeneous [18]. Meta-ANOVA and meta-regression were performed for the moderating variables, and both continuous variables (sample sizes) and categorical variables (gender, age, study period, publication type) were estimated using the Restricted Maximum Likelihood (REML) method [19].

### 2.6. Assessment of Heterogeneity

Heterogeneity was assessed using the Cochrane Q test and Higgins’ I^2^ statistics. When the *p*-value of the Cochrane Q test was 0.1 or less or the Higgins’ I^2^ was 50% or more, it was considered that there was statistically significant heterogeneity.

### 2.7. Publication Bias

For the publication bias analysis, we applied a funnel plot using standard error and MD, additionally applied Egger’s test and Begg’s test [20].

## 3. Result

### 3.1. Inclusion of Studies

**Figure 1:**
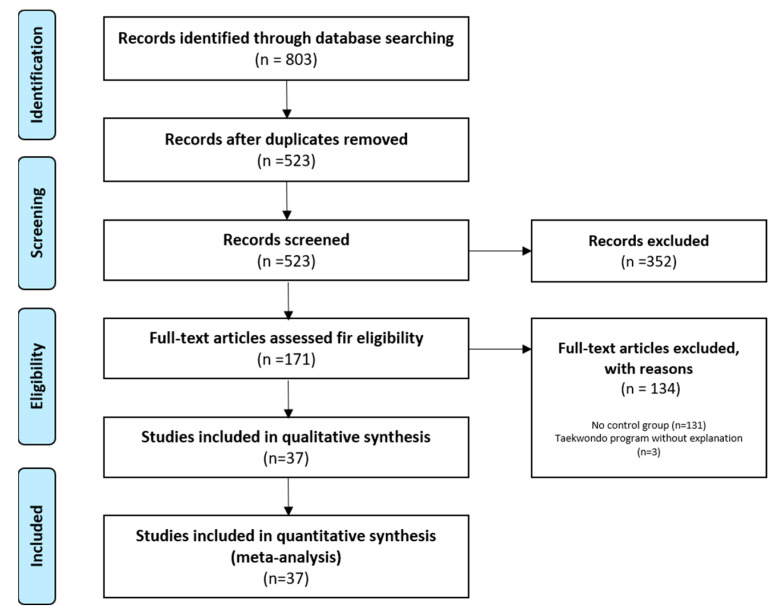
PRISMA flow diagram.

Of the 803 studies, we selected 171 studies, excluding duplicate studies (280) and those unrelated to this study (352). Then, as a result of full-text screening according to the PICO criteria, 131 studies without a control group and three studies that did not explain the Taekwondo training program were excluded. Therefore, we finally selected 37 studies for meta-analysis. All 37 papers were satisfied for both qualitative and quantitative analysis. The detailed process is shown in Figure 1.

### 3.2. Characteristics of the Included Studies

The total sample size was 801. The average age of the participants was 32.19 years old, ranging from 7.46 to 75.07, and 54.2% of the participants were male. 18 studies [21,22,23,24,25,26,27,28,29,30,31,32,33,34,35,36,37] were for students, 12 studies [38,39,40,41,42,43,44,45,46,47,48,49] were for adults, and 7 studies were for seniors [50,51,52,53,54,55,56], and the training period was 8–24 months. According to the publication type, 15 were for dissertations studies, and 22 studies were for journal publication. The characteristics of the included studies are summarized in Table 1.

### 3.3. Quality Assessment

**Figure 2:**
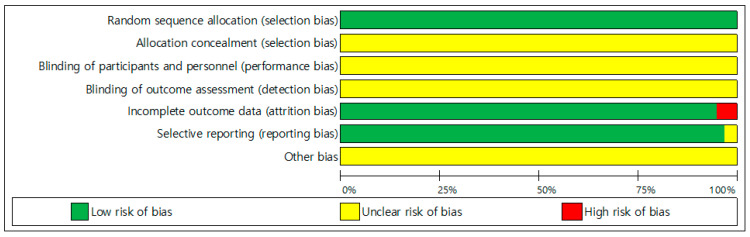
Assessment of risk of bias in included studies.

Two independent researchers (S.B., S.N.) performed quality assessment as shown in Figure 2, based on the criteria of the Cochrane Collaboration. All 37 studies were randomized control studies, and all studies analyzed the results, excluding participants who dropped out during the intervention.

### 3.4. Outcome Findings

#### 3.4.1. Height

**Figure 3:**
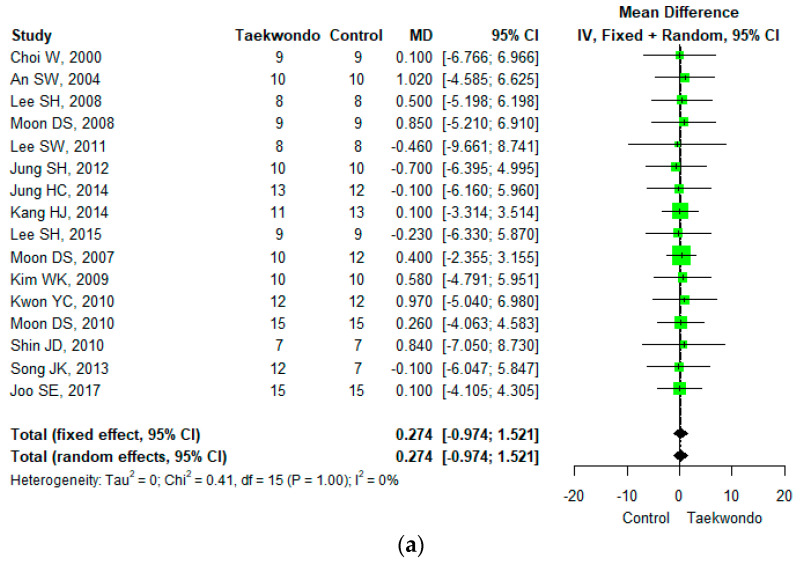

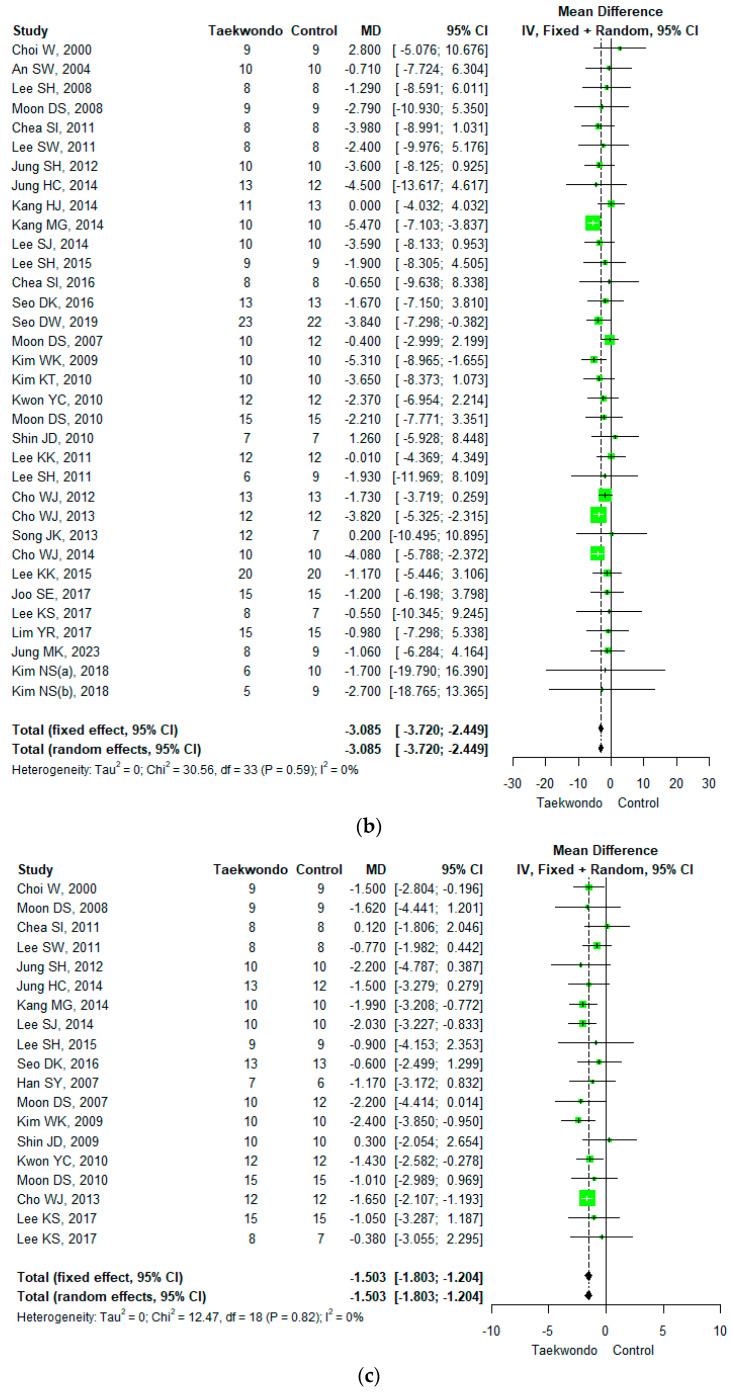

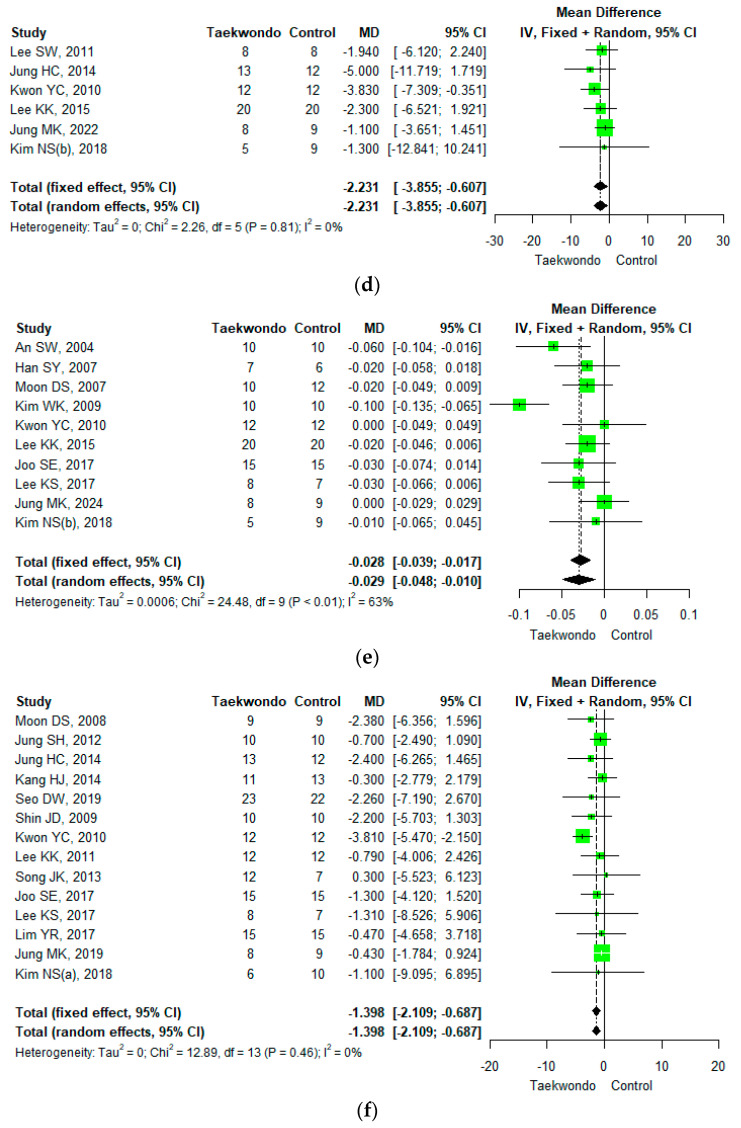

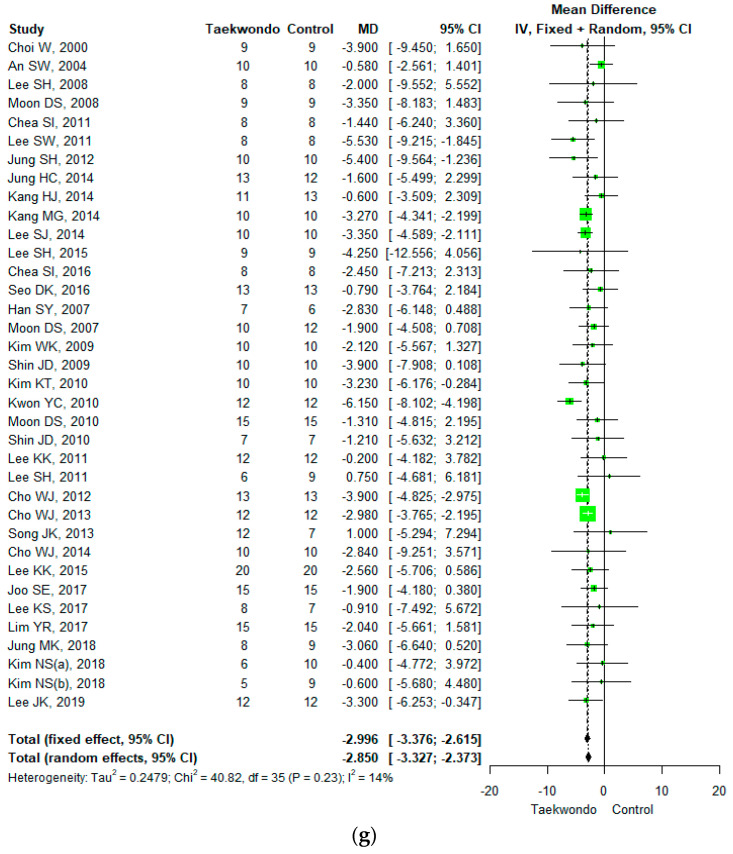

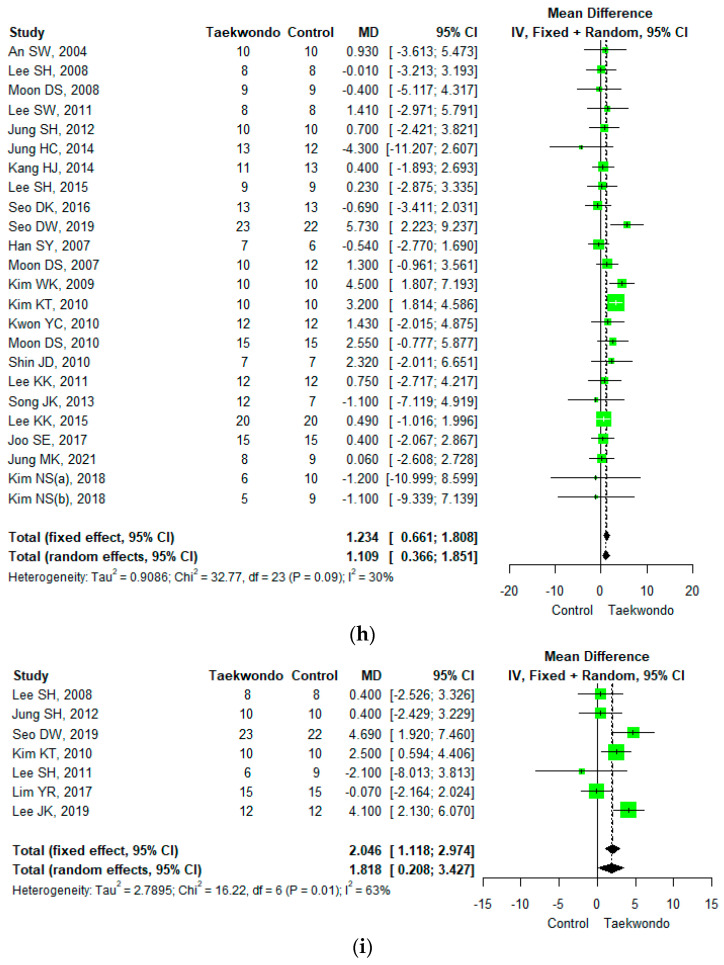
Forest plot of the mean difference in physique and body composition. (**a**) Height; (**b**) weight; (**c**) body mass index; (**d**) waist circumference; (**e**) waist-hip ratio; (**f**) body fat mass; (**g**) body fat percentage; (**h**) lean mass; (**i**) muscle mass.

Sixteen studies reported height (total *n* = 184; taekwondo training group *n* = 168, control group *n* = 166). The mean difference (MD) in heights between the Taekwondo training and the control group was 0.274 (95% CI; −0.974, 1.521), and no statistically significant difference was found. For the heterogeneity test, the results of Cochrane Q were *p* = 1.00, and Higgins’ I^2^ was 0% (Figure 3a).

#### 3.4.2. Weight

A total of 34 studies reported body weight (total *n* = 738; taekwondo training group *n*= 365, control group *n* = 373). The mean difference (MD) in body weight between the Taekwondo training and the control group was −3.085 (95% CI; −3.720, −2.449). That is, the weight of the Taekwondo training group decreased by 3.1 kg compared to the control group, which was statistically significant (*p* < 0.001). For the heterogeneity test, the results of Cochrane Q were *p* = 0.59, and Higgins’ I^2^ was 0% (Figure 3b).

#### 3.4.3. Body Mass Index

Eighteen studies reported body mass index (total *n* = 395; 198 Taekwondo training group, 197 control group). The mean difference (MD) of body mass index between the Taekwondo training and the control group was −1.503 (95% CI; −1.803, −1.204). The Body mass index of the Taekwondo training group decreased by 1.5 compared to the control group, and there was a statistically significant difference (*p* < 0.001). For the heterogeneity test, the results of Cochrane Q were *p* = 0.82, and Higgins’ I^2^ was 0% (Figure 3c).

#### 3.4.4. Waist Circumference

Six studies reported waist circumference (total *n* = 136; taekwondo training group *n* = 66, control group *n* = 70). The mean difference (MD) in waist circumference between the Taekwondo training and the control group was −2.231 (95% CI; −3.855, −0.607). The waist circumference of the Taekwondo training group decreased by 2.2 cm compared to the control group, and there was a statistically significant difference (*p* < 0.05). For the heterogeneity test, the results of Cochrane Q were *p* = 0.81, and Higgins’ I^2^ was 0% (Figure 3d).

#### 3.4.5. Waist–Hip Ratio

Ten studies reported the waist–hip ratio (total *n* = 215; Taekwondo training group *n*= 105, control group *n* = 110). The mean difference (MD) in the waist–hip ratio between the Taekwondo training and the control groups was −0.029 (95% CI; −0.015, −0.010). The waist–hip ratio of the Taekwondo training group decreased by 0.03 compared to the control group, and there was a statistically significant difference (*p* < 0.05). For the heterogeneity test, the results of Cochrane Q were *p* <0.01, and Higgins’ I^2^ was 0% (Figure 3e).

#### 3.4.6. Body Fat Mass

Fourteen studies reported body fat mass (total *n* = 327; Taekwondo training group *n* = 164, the control group *n* = 163). The average difference (MD) in body fat mass between the Taekwondo training and the control group was −1.398 (95% CI; −2.109, −0.687). The body fat mass of the Taekwondo training group decreased by 1.40 kg compared to the control group, and there was a statistically significant difference (*p* < 0.01). For the heterogeneity test, the results of Cochrane Q were *p* = 0.46, and Higgins’ I^2^ was 0% (Figure 3f).

#### 3.4.7. Body Fat Percentage

Thirty-six studies reported body fat percentage (total *n* = 750; taekwondo training group *n*= 371, control group *n* = 379). The mean difference (MD) in body fat percentage between the Taekwondo training and the control group was −2.850 (95% CI; −3.327, −2.373). The body fat percentage of the Taekwondo training group decreased by −2.90% compared to the control group, and there was a statistically significant difference (*p* < 0.001). For the heterogeneity test, the results of Cochrane Q were *p* = 0.23, and Higgins’ I^2^ was 14% (Figure 3g).

#### 3.4.8. Lean Mass

Twenty-four studies reported lean mass (total *n* = 531; taekwondo training group *n* = 263, control group *n* = 268). The average difference (MD) in lean mass between the Taekwondo training and the control groups was 1.109 (95 % CI; 0.366, 1.851). The lean mass of the Taekwondo training group increased by 1.11 kg compared to the control group, and there was a statistically significant difference (*p* < 0.05). For the heterogeneity test, the results of Cochrane Q were *p* = 0.09, and Higgins’ I^2^ was 30% (Figure 3h).

#### 3.4.9. Muscle Mass

Seven studies reported muscle mass (total *n* = 170; Taekwondo training group *n* = 84, control group *n* = 86). The mean difference (MD) in muscle mass between the Taekwondo training and the control group was 1.818 (95% CI; 0.208, 3.427). The muscle mass of the Taekwondo training group increased by 1.82 kg compared to the control group, and there was a statistically significant difference (*p* < 0.05). For the heterogeneity test, the results of Cochrane Q were *p* = 0.01, and Higgins’ I^2^ was 63% (Figure 3i).

### 3.5. Moderator Analyses

The results of meta-ANOVA with categorical variables (gender, age, study period, publication type) are shown in Table 2 and Table 3. No statistical significance was reported on moderating variables of height, WC, WHR, body fat mass, lean mass, and muscle mass. Meanwhile, in the case of body weight (*p* = 0.044), BMI (*p* = 0.036), and body fat percentage (*p* = 0.026), the age group was confirmed as a statistically significant moderating variable. It was confirmed that the decrease rate of weight and BMI was the highest among the student group, and the decrease rate of body fat percentage was highest among the elderly group.

### 3.6. Publication Bias

**Figure 4:**
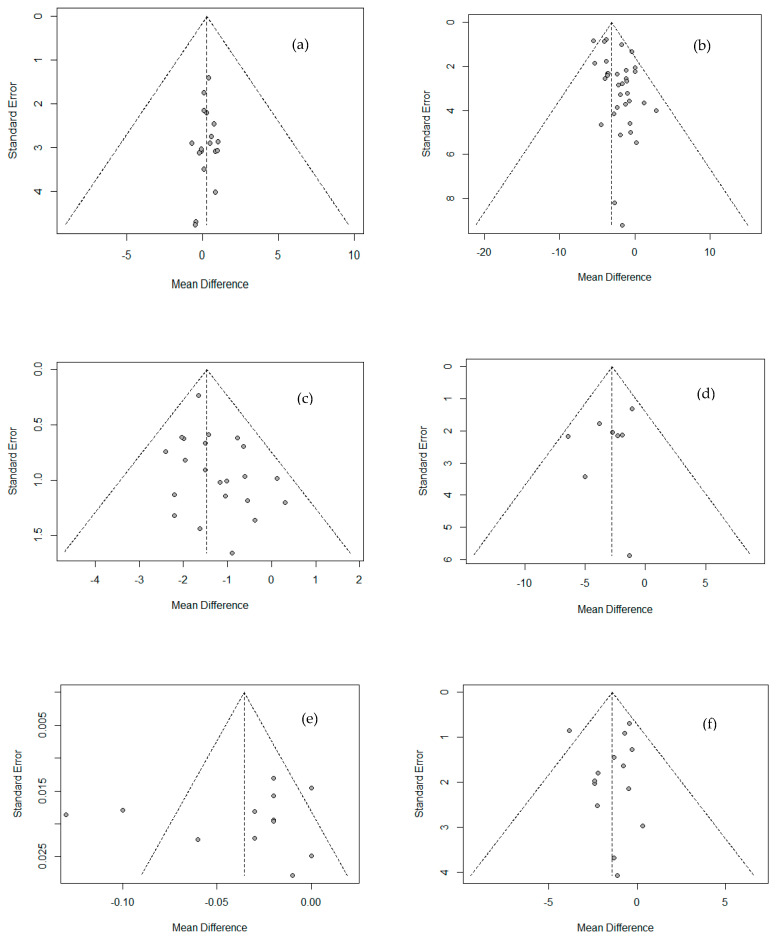

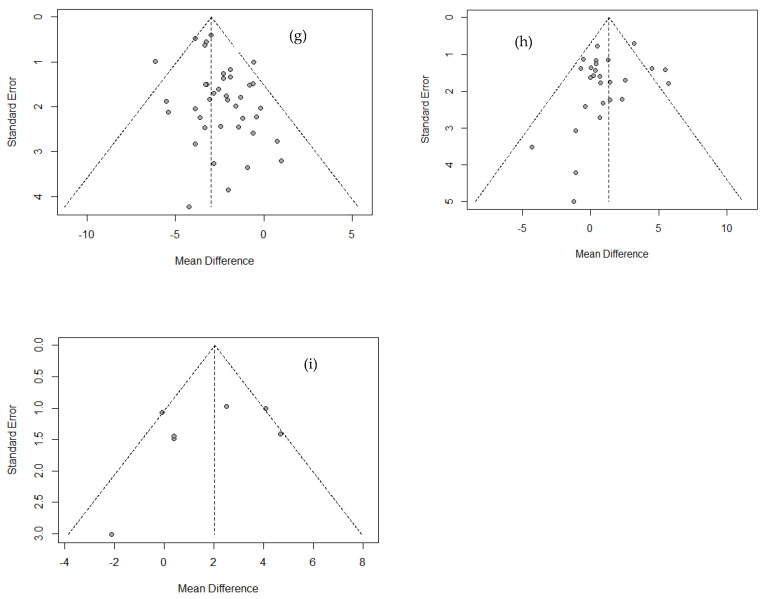
Funnel plot showing the publication bias of Physique and body composition. (**a**) Height; (**b**) weight; (**c**) BMI; (**d**) WC; (**e**) WHR; (**f**) fat mass.; (**g**) % body fat; (**h**) lean mass; (**i**) muscle mass.

Funnel plots were used to analyze publication bias or effects of small-sized research, as shown in Figure 4. No publication bias was found from Egger’s linear regression test among all variables except weight and body fat percentage, excluding small-sized studies (WC, muscle mass). Also, as a result of Begg’s test using a rank correlation test, no factor showed a significant difference, so the publication bias of the literature used in this study could not be confirmed.

## 4. Discussion

Since changes in body composition occur throughout the lifespan due to growth, maturation and aging, as well as factors such as diseases and behaviors, it is a representative indicator for determining the level of body development [58]. Observing changes in body composition is an important factor in determining health, disease, exercise, and nutrition. It is also a very important factor in physique and athletic performance [59].

In general, Taekwondo training in Korea consists of 5 sessions per week, 1 h per session [60]. The training period is about 12 weeks, which we present in Table 1. Taekwondo exercise consists of 5 min each of warm-up and cool-down routines, followed by 50 min of exercise including basic Taekwondo movements, Poomsae, and Gyeorugi (fighting simulation) as the main exercises. Most taekwondo training centers apply a similar method. Therefore, the effect of taekwondo training in this study has very suitable conditions for meta-analysis.

As a result of analyzing the effect of Taekwondo training on changes in body composition, statistically significant differences were found in body weight, BMI, WC, body fat mass, body fat percentage, lean mass, and muscle mass, excluding height. Especially in the case of students, body weight and body fat percentage decreased significantly, and lean mass and muscle mass also tended to increase. In the elderly, a significant decrease in body fat percentage and an increase in muscle mass were found.

According to ACSM guidelines, exercise intensity 40–70% VO_2_R (oxygen uptake reserve) is recommended for overweight and obesity management [61]. Taekwondo’s basic movements, kicks, and Poomsae movements have an exercise intensity of 84% HRmax and 56.8~82.2% VO_2_max [62]. Taekwondo competition is a high-intensity exercise with an exercise intensity of 10 METs [63]. As such, Taekwondo exhibits anaerobic exercise patterns during matches and competitions and aerobic exercise patterns during taekwondo aerobics, Poomsae, sparring steps, basic movements, and moving kicks.

Therefore, the results of this study are clear evidence that the type of exercise and intensity of Taekwondo has positive effects on reducing body weight and body fat and increasing lean mass. In addition, there is a disadvantage that long and tedious exercise time is required to obtain meaningful exercise effects with general aerobic exercise such as walking or running [64]. In contrast, Taekwondo is an interval training type that alternates high- and low-intensity exercise repeatedly. In addition, Taekwondo gives interest and has higher exercise effects within the same amount of exercise time than other exercise types such as walking or moderate running [65]. Therefore, the exercise program using Taekwondo will positively improve obesity, the most severe health issue in Korea, and contribute significantly to preventing chronic diseases such as cardiovascular disease.

We found one unfortunate thing while conducting this study. That is, many studies reporting on the effectiveness of Taekwondo training do not apply an RCT design or even have no control group. In fact, there were many documents excluded from the analysis because there was no control group during this study. Therefore, if researchers who want to explain the effects of Taekwondo training in the future follow the RCT design well, follow-up researchers will find clearer results.

## 5. Conclusions

We found that taekwondo training at a frequency of five times per week for more than 12 weeks positively improved the obesity factor through this study. These findings are clear evidence that Taekwondo training is an effective exercise that can prevent or positively improve obesity. In addition, it shows that Taekwondo has value as a lifestyle sport that can contribute to the promotion of human health, not just bounded in the field of martial arts and sports.

## Figures and Tables

**Table 1:** Characteristics of included studies.

Study	Study Type	Frequency (Day/Week)	Participants	Sex	Taekwondo (*n*, Age)		Control (*n*, Age)		Body Composition Method	Outcome Variable
Choi W, 2000 [21]	Thesis	3/24	Elementary student, 40% body fat ↑	M	9	11.78	9	11.89	Skinfold	% Body fat, BMI, Height, Weight
Lee SH, 2008 [22]	Thesis	5/12	Elementary student	M	8	11.1	8	11.1	BIA	% Body fat, Height, Lean mass, Muscle mass, Weight
Lee SW, 2011 [23]	Thesis	5/12	Elementary student	Mixed	8	11.13	8	10.88	BIA	% Body fat, BMI, Height, Lean mass, WC, Weight
Jung SH, 2012 [24]	Thesis	5/12	Elementary student	M	10	10.2	10	10.3	BIA	% Body fat, BMI, Fat mass, Height, Lean mass, Muscle mass, Weight
Lee SJ, 2014 [25]	Thesis	3/16	Elementary student, 25% body fat ↑	M	10	12.6	10	12.7	BIA	% Body fat, BMI, Weight
Lee SH, 2015 [26]	Thesis	5/12	Elementary student	M	9	7.46	9	7.74	BIA	% Body fat, BMI, Height, Lean mass, Weight
Seo DW, 2019 [27]	Thesis	5/12	Elementary student	M	23	10	22	9.67	BIA	Fat mass, Lean mass, Muscle mass, Weight
An SW, 2004 [28]	Thesis	5/12	Adolescent, 30% body fat ↑	M	10	ND	10	ND	BIA	% Body fat, Height, Lean mass, Weight, WHR
Shu DK, 2008 [29]	Thesis	5/12	Adolescent. first menstruation	M	9	12.18	9	12.73	BIA	% Body fat, BMI, Fat mass, Height, Lean mass, Weight
Jung HC, 2014 [30]	Thesis	3/16	Adolescent	M	15	13.9	15	13.9	DXA	% Body fat, BMI, Fat mass, Height, Lean mass, WC, Weight
Kang MG, 2014 [31]	Thesis	3/12	Adolescent, 30% body fat ↑	F	10	ND	10	ND	BIA	% Body fat, BMI, Weight
Moon DS, 2007 [32]	Journal article	5/12	Elementary student	M	12	12.35	12	12.42	BIA	% Body fat, BMI, Height, Lean mass, Weight, WHR
Kim WK, 2009 [33]	Journal article	5/12	Adolescent men, 20% body fat ↑	M	10	14.7	10	15.1	BIA	% Body fat, BMI, Height, Lean mass, Weight, WHR
Kwon YC, 2010 [34]	Journal article	3/12	Elementary student, 25% body fat ↑	Mixed	12	11.92	12	12.5	BIA	% Body fat, BMI, Fat mass, Height, Lean mass, WC, Weight, WHR
Lee SH, 2011 [35]	Journal article	4/10	Adolescent	M	6	16.8	9	16.4	BIA	% Body fat, Muscle mass, Weight
Cho WJ, 2013 [36]	Journal article	3/12	Elementary student, BMI 25 kg/m^2^ ↑	M	12	11.17	12	11.33	BIA	% Body fat, BMI, Weight
Song JK, 2013 [37]	Journal article	3/12	Adolescent	M	12	14	7	13.9	DXA	% Body fat, Fat mass, Height, Lean mass, Weight
Cho WJ, 2014 [38]	Journal article	3/12	Elementary student, BMI 25 kg/m^2^ ↑	M	10	11.77	10	11.51	BIA	% Body fat, Weight
Chea SI, 2016 [39]	Thesis	3/12	Middle-aged women, obesity	F	8	39.25	8	39.7	BIA	% Body fat, Weight
Seo DK, 2016 [40]	Thesis	5/12	Middle-aged women	F	13	42.77	13	42.54	BIA	% Body fat, BMI, Lean mass, Weight
Han SY, 2007 [41]	Journal article	5/14	Middle-aged women, no menopause	F	7	41	6	38	BIA	% Body fat, BMI, Lean mass, WHR
Kim KT, 2010 [42]	Journal article	3/12	Adult	M	10	27.3	10	27.4	BIA	% Body fat, Lean mass, Muscle mass, Weight
Lee KK, 2011 [43]	Journal article	3/12	Middle-aged	F	12	41.44	12	42.16	DXA	% Body fat, Fat mass, Lean mass, Weight
Lee KK, 2015 [44]	Journal article	3/12	Middle-aged women, menopause	F	20	54.3	20	53.1	DXA	% Body fat, Lean mass, WC, Weight, WHR
Joo SE, 2017 [45]	Journal article	3/12	Middle-aged women, 30% body fat ↑	F	15	40.1	15	40.2	BIA	% Body fat, Fat mass, Height, Lean mass, Weight, WHR
Lee KS, 2017 [46]	Journal article	3/8	Middle-aged	M	7	40.85	7	40.71	BIA	% Body fat, BMI, BMI, Fat mass, Weight, WHR
Jung MK, 2018 [47]	Journal article	3/24	Middle-aged women, menopause & obese	F	8	61.05	9	59.89	BIA	% Body fat, Fat mass, Lean mass, WC, Weight, WHR
Lee JK, 2019 [48]	Journal article	3/12	Middle-aged women, 30% body fat ↑	F	12	50.9	12	50.3	BIA	% Body fat, Muscle mass
Kim NS, 2018 [49]	Journal article	13/2	University student	M	6	21.3	10	22.1	BIA	% Body fat, Fat mass, Lean mass, Weight
Kim NS, 2018 [50]	Journal article	3/12	University student	M	5	21	9	22	BIA	% Body fat, Lean mass, WC, Weight, WHR
Chea SI, 2011 [51]	Thesis	3/12	Elderly women	F	8	69.53	8	70	BIA	% Body fat, BMI, Weight
Kang HJ, 2014 [52]	Thesis	3/12	Elderly women	F	11	69.4	13	70.4	DXA	% Body fat, Fat mass, Height, Lean mass, Weight
Shin JD, 2009 [53]	Journal article	3/12	Elderly women	F	10	69.7	10	71.7	BIA	% Body fat, BMI, Fat mass,
Moon DS, 2010 [54]	Journal article	3/12	Elderly women	F	15	72.13	15	75.07	BIA	% Body fat, BMI, Height, Lean mass, Weight
Shin JD, 2010 [55]	Journal article	3/12	Elderly women, 30% body fat ↑	F	7	70.86	7	71.68	BIA	% Body fat, Height, Lean mass, Weight
Cho WJ, 2012 [56]	Journal article	3/12	Elderly women, 30% body fat ↑	F	13	69	13	68.62	BIA	% Body fat, Weight
Lim YR, 2017 [57]	Journal article	4/12	Elderly women, over Weight	F	15	ND	15	ND	BIA	% Body fat, Fat mass, Muscle mass, Weight

**Table 2:** Effects of moderators on height, weight, BMI, WC, WHR.

	Height					Weight					BMI					Waist Circumference					WHR				
Variables	*k*	MD	95% CI		*p* ^†^	*k*	MD	95% CI		*p* ^†^	*k*	MD	95% CI		*p* ^†^	*k*	MD	95% CI		*p* ^†^	*k*	MD	95% CI		*p* ^†^
Sex					0.985					0.154					0.100					0.404					0.111
Male	9	0.246	−1.412	1.905		18	−3.314	−4.171	−2.455		9	−1.708	−2.074	−1.342		2	−4.063	−9.870	1.743		5	−0.045	−0.078	−0.012	
Female	5	0.271	−1.774	2.136		14	−2.147	−3.505	−0.790		8	−1.068	−1.738	−0.399		2	−1.421	−3.605	0.762		4	−0.015	−0.031	−0.001	
Age					0.988					0.044					0.036					0.473					0.192
Student	12	0.312	−1.213	1.836		18	−3.748	−4.503	−2.992		12	−1.657	−1.981	−1.334		3	−3.322	−5.807	−0.838		4	−0.046	−0.090	−0.002	
Adult	2	0.1	−2.55	2.75		11	−1.129	−2.839	0.582		3	−0.243	−1.434	0.948		2	−2.182	−6.147	1.783		5	−0.023	−0.039	−0.006	
Elderly	2	0.394	−3.397	4.185		6	−1.775	−3.333	−0.217		4	−0.832	−1.907	0.242		1	−1.100	−3.651	1.451		1	−0.000	−0.029	0.029	
Study duration					0.898					0.689					0.542					0.405					-
≤12 weeks	14	0.297	−1.001	1.595		31	−3.107	−3.753	−2.462		16	−1.465	−1.789	−1.141		5	−2.059	−3.732	−0.386		10	−0.029	−0.048	−0.010	
>12 weeks	2	−0.012	−4.556	4.531		3	−2.315	−6.143	1.513		3	−1.731	−2.521	−0.941		1	−5.000	−11.719	1.719		-	-	-	-	
Publication type					0.865					0.132					0.593					0.726					0.169
Thesis	9	0.156	−1.689	2.001		15	−3.775	−4.874	−2.675		10	−1.392	−1.9	−0.884		2	−2.794	−6.343	0.756		1	−0.060	−0.104	−0.016	
Journal	7	0.373	−0.321	2.067		19	−2.739	−3.518	−1.960		9	−1.563	−1.935	−1.192		4	−2.082	−3.908	−0.256		9	−0.026	−0.046	−0.006	

MD—Mean difference by the Hedges. *k*—number of observations. ^†^ *p*-value of meta-ANOVA for categorical moderators.

**Table 3:** Effects of moderators on fat mass, % fat, lean mass, muscle mass.

	Fat Mass					% Body Fat					Lean Mass					Muscle Mass				
Variables	*k*	MD	95% CI		*p* ^†^	*k*	MD	95% CI		*p* ^†^	*k*	MD	95% CI		*p* ^†^	*k*	MD	95% CI		*p* ^†^
Sex					0.761					0.404					0.088					0.896
Male	6	−1.043	−2.480	0.393		17	−2.641	−3.228	−2.121		12	1.718	0.388	3.048		5	1.733	−0.125	3.59	
Female	7	−0.777	−1.717	0.163		17	−3.006	−3.556	−2.457		10	0.364	−0.440	1.169		2	2.031	−2.056	6.117	
Age					0.240					0.026					0.700					0.621
Student	6	−2.180	−3.614	−0.745		17	−3.047	−3.842	−2.304		12	1.419	0.152	2.686		4	1.313	−1.336	3.962	
Adult	6	−0.750	−2.198	0.698		13	−1.867	−2.802	−0.931		9	0.715	−0.420	1.850		3	2.201	−0.12	4.522	
Elderly	2	−0.660	−1.923	0.603		6	−3.549	−4.370	−2.728		3	1.276	−0.600	3.151		-	-	-	-	
Study duration					0.610					0.475					0.122					
≤12 weeks	13	−1.370	−2.134	−0.606		33	−2.756	−3.298	−2.213		23	1.173	0.447	1.900		7	1.818	0.208	3.427	
>12 weeks	1	−2.400	−6.265	1.465		3	−3.220	−4.375	−2.065		1	−4.300	−11.207	2.607		-	-	-	-	
Publication type					0.544					0.627					0.132					0.952
Thesis	5	−1.039	−2.283	0.205		14	−2.673	−3.457	−1.889		15	−3.775	−4.874	−2.675		3	1.854	−0.984	4.692	
Journal	9	−1.572	−2.438	−0.706		22	−2.923	−3.551	−2.295		19	−2.739	−3.518	−1.960		4	1.742	−0.522	4.007	

MD—Mean difference by the Hedges. *k*—number of observations. ^†^ *p*-value of meta-ANOVA for categorical moderators.

## Data Availability

Not applicable.
